# Management of Atrio-esophageal Fistula Induced by Radiofrequency Catheter Ablation in Atrial Fibrillation Patients: a Case Series

**DOI:** 10.1038/s41598-020-65185-9

**Published:** 2020-05-18

**Authors:** Yun Gi Kim, Jaemin Shim, Kwang-No Lee, Ju Yong Lim, Jae Ho Chung, Jae Seung Jung, Jong-Il Choi, Sung Ho Lee, Ho Sung Son, Young-Hoon Kim

**Affiliations:** 1 Arrhythmia Center, Korea University Medicine Anam Hospital, Seoul, Republic of Korea; 2 Department of Thoracic and Cardiovascular Surgery, Korea University Medicine Anam Hospital, Seoul, Republic of Korea

**Keywords:** Cardiology, Risk factors

## Abstract

Atrio-esophageal fistula (AEF) is one of the most devastating complication of radiofrequency catheter ablation (RFCA) of atrial fibrillation (AF) and surgical repair is strongly recommended. However, optimal surgical approach remains to be elucidated. We retrospectively reviewed AEF cases that occurred after RFCA in a single center and evaluated the clinical results of different surgical approach. Surgical or endoscopic repair was attempted in five AF patients who underwent RFCA. Atrio-esophageal fistula and mediastinal infection was not controlled in the patient who underwent endoscopic repair eventually died. Lethal cerebral air embolism occurred two days after surgery in a patient who underwent esophageal repair only. Primary surgical repair of both the left atrium (LA) and esophagus was performed in the remaining three patients. Among these three patients, two underwent external LA repair and the remaining had internal LA repair via open-heart surgery. External repair of the LA was unsuccessful and one patient dies and another had to undergo second operation with internal repair of the LA. The patient who underwent internal LA repair during the first operation survived without additional surgery. Furthermore, we applied veno-arterial extracorporeal membrane oxygenation (VA-ECMO) with artificial induction of ventricular fibrillation in this patient to prevent air and septic embolism and she had no neurologic sequelae. In summary, surgical correction can be considered preferentially to correct AEF. Open-heart surgical repair of LA from the internal side seems to be an acceptable surgical method. Application of VA-ECMO with artificial induction of ventricular fibrillation might be effective to prevent air and septic embolism.

## Introduction

The number of radiofrequency catheter ablation (RFCA) procedures to treat atrial fibrillation (AF) is increasing rapidly largely due to recent advancements in the procedural outcomes and enlarging overall burden of AF^1,2^. RFCA is associated with significant improvements in maintenance of sinus rhythm and the quality of life^2,3^. The CASTLE-AF trial demonstrated that RFCA is associated with increased overall survival in AF patients with advanced heart failure and the CABANA trial suggested a potential benefit in major cardiovascular events, although limited to per-protocol and as-treated analysis, in general AF patients^4,5^. However, RFCA is also associated with life threatening or disabling complications, such as cardiac tamponade, procedure-related ischemic stroke, stiff left atrium (LA) syndrome, and atrio-esophageal fistula (AEF)^6–9^.

Among various complications of RFCA, AEF is probably the most catastrophic one due to several reasons. The mortality rate is high and might exceed 80% in severe cases^7,10^. The onset is delayed and usually occurs several days or weeks after the procedure which impedes prompt diagnosis and early intervention^4,11^. Presenting symptoms such as fever, chilling, cough, or neurologic symptoms are also far from specific further hindering timely diagnosis^7,12^. The absence of validated method to prevent AEF is another challenge for interventional electrophysiologists^13^. However, top list among various unmet needs regarding AEF is probably how to save lives of these patients without neurologic consequences. Unfortunately, there is no validated or standardized method to manage patients with AEF. Atrio-esophageal fistula induced by RFCA is largely thermal and ischemic injury and therefore, is different from other type of injuries such as mechanical injury^14^. The repair of the esophagus is difficult due to necrosis of surrounding tissues and thus, surgical techniques should be aggressive and sophisticated. We reviewed our prior RFCA-related AEF cases who underwent surgery or endoscopic repair and aimed to broaden our knowledge on how to save lives of patients with RFCA-related AEF without neurologic consequences.

## Methods

### Patients

Consecutive AF patients undergoing RFCA at Korea University Medical Center Anam Hospital between June 1998 and December 2019 were analyzed retrospectively. All (N = 4,009) patients who underwent RFCA in our institution were screened and there were no specific exclusion criteria. Patients who developed AEF and underwent surgical or endoscopic repair were enrolled. The Institutional Review Board of Korea University Medical Center Anam Hospital approved this study and written informed consent was waived because the current study was a retrospective analysis. The study protocol adheres to the ethical guidelines of the 2008 Declaration of Helsinki.

### Ablation procedure

The precise protocol for RFCA at our institution is published elsewhere^7,9,15^. Barium esophagography was performed to visualize the location of esophagus immediately before sedative infusion. Fluoroscopy of the chest was taken after swallowing the barium contrast in RAO, AP, and LAO views to visualize the location of esophagus. After positioning circular mapping catheter and ablation catheter, LA geometry and ablation were performed. For 3 dimensional mapping system, either EnSite NavX/Velocity (St. Jude Medical, St. Paul, Minnesota) or CARTO (Biosense Webster, Irvine, California) systems were used. Barium esophagography-guided catheter ablation of posterior aspect of left pulmonary veins was performed to minimize direct contact of ablation catheter with the esophagus and to avoid excessive energy delivery near the esophagus. Left-sided posterior wall was ablated with 25 W to minimize esophageal injury and other areas were ablated with 30 to 35 W.

### Diagnosis of AEF

The diagnosis of AEF was made when a patient had compatible symptoms and had air leakage into LA in imaging studies such as computed tomography (CT) or magnetic resonance imaging (MRI). Electrophysiologist and radiologist worked together to make correct diagnosis.

### Data availability

All data generated or analyzed during this study are included in this published article (and its Supplementary Information files).

## Results

### AEF cases

A total of five patients suffered from AEF and underwent either surgical or endoscopic repair. Baseline characteristics and brief history of these patients are summarized in Table 1. All five patients had air leakage into LA or pericardial space. Mean age was 60.0 ± 3.1 and three patients were male (60.0%). All patients underwent wide antral circumferential pulmonary vein isolation. Non-pulmonary vein trigger foci ablation, complex fractionated atrial electrogram guided ablation, and cavotricuspid isthmus ablation was performed in one, one, and three patients, respectively. Three patients eventually died: one patient due to septic shock during recovery phase after corrective surgery, another patient due to uncontrolled infection and cachexia, and the last patient due to massive cerebral air embolism.

**Table 1:** Baseline demographics.

	Age	Sex	CHA_2_DS_2_-VASc	Time to symptom onset (days)	Time to intervention (days)	Ablation catheter	Ablation power during posterior wall ablation	Lesion set
Patient 1	55	Male	0	32	32	Non-contact force Open-irrigation	25 W	PVI + non-PV trigger foci (interatrial septum) + CTI
Patient 2	64	Male	1	27	29	Non-contact force Open-irrigation	25 W	PVI
Patient 3	60	Female	3	8	8	Non-contact force Open-irrigation	25 W	PVI
Patient 4	57	Male	1	25	26	Non-contact force Open-irrigation	25 W	PVI + CTI
Patient 5	58	Female	2	25	27	Non-contact force Open-irrigation	25 W	PVI + CFAE (LA anterior wall, lateral perimitral isthmus, crista terminalis, coronary sinus ostium) + CTI

CFAE: complex fractionated atrial electrogram; CTI: cavotricuspid isthmus; LA: left atrium; PV: pulmonary vein; PVI: pulmonary vein isolation.

### Repair of AEF

**Figure 1:**
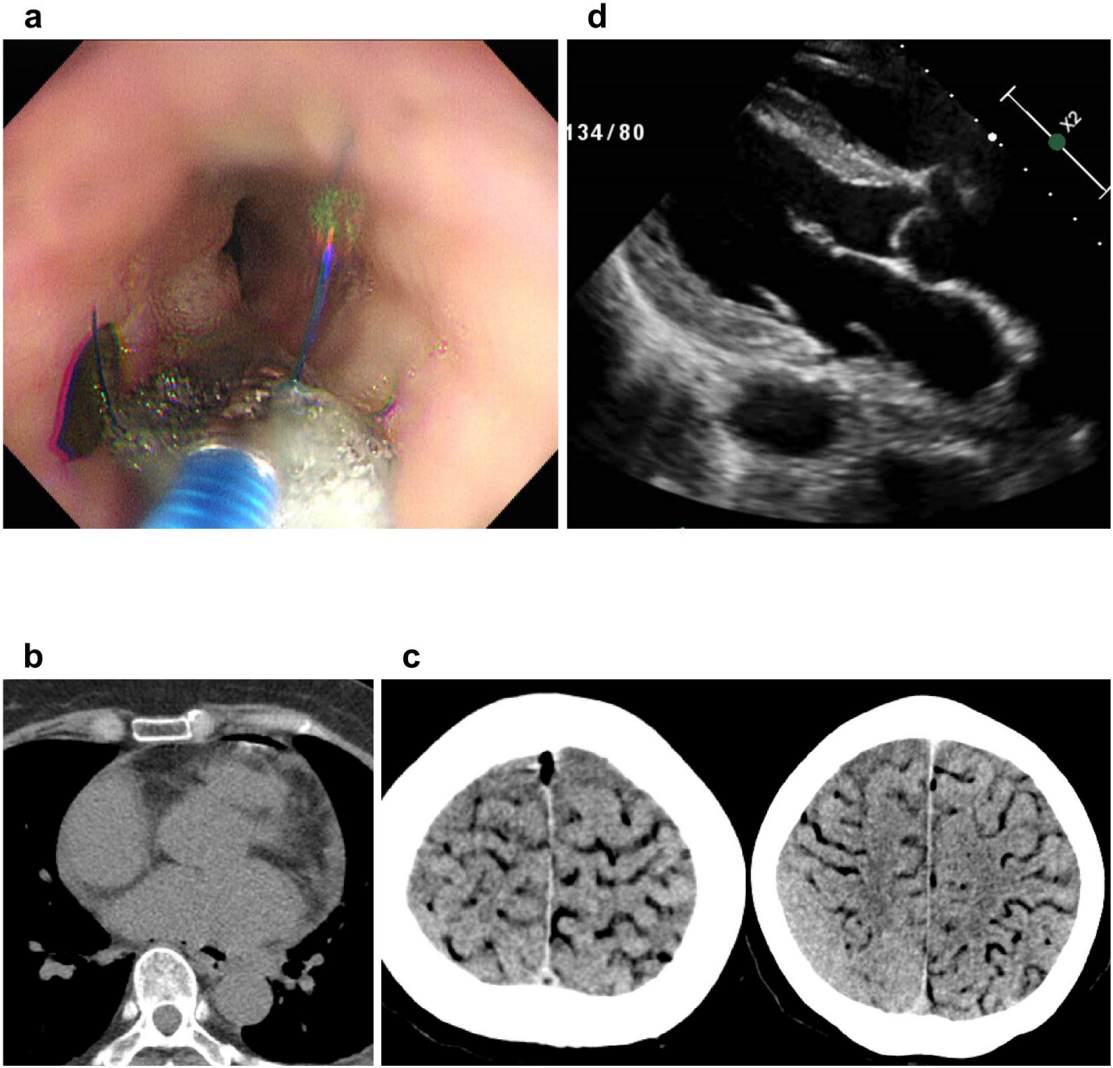
Unsuccessful approaches for atrio-esophageal fistula. (**a**) Multiple endoscopic repair attempts using sponge material failed to heal atrio-esophageal fistula. (**b**) Primary repair of the LA from the external side failed to heal LA perforation lesion. Large vegetation originating from LA perforation site is observed in echocardiography. Patient underwent on-pump, open heart redo surgical correction of the LA which was successful. (**c** and **d**) The patient suffered massive cerebral air embolism after primary esophageal repair without LA repair. LA: left atrium.

All five patients underwent either surgical or endoscopic repair of AEF: four patients underwent surgery and one patient endoscopic repair. AEF was not repaired in the patient who underwent endoscopic repair and he eventually died due to uncontrolled infection and cachexia (Fig. 1a). Esophageal fistulectomy and primary repair with intercostal muscle flap interposition was performed in all four patients who underwent surgical repair. However, they had different surgical approach to repair LA defect (Table 1). Patient 1 underwent off-pump LA repair from external side with bovine pericardium patch support. Patient 3 did not undergo LA repair because CT scan showed no free air in the LA and no LA defect was observed during operation (Fig. 1b). The patient had a coughing event which led to massive cerebral air embolism two days after the surgery (Fig. 1c). Patient 4 had off-pump LA repair from external side without bovine pericardium patch support. Due to subsequent endocarditis with large floating vegetation located in the LA defect site (Supplementary Video 1), the patient had to undergo redo on-pump open heart surgery with LA repair from inside of the heart with bovine pericardium patch support (Fig. 1d). Patient 5 underwent on-pump LA repair from internal side with bovine pericardial patch support. The esophagus and LA healed successfully without any additional surgery.

### VA-ECMO and induction of VF

**Figure 2:**
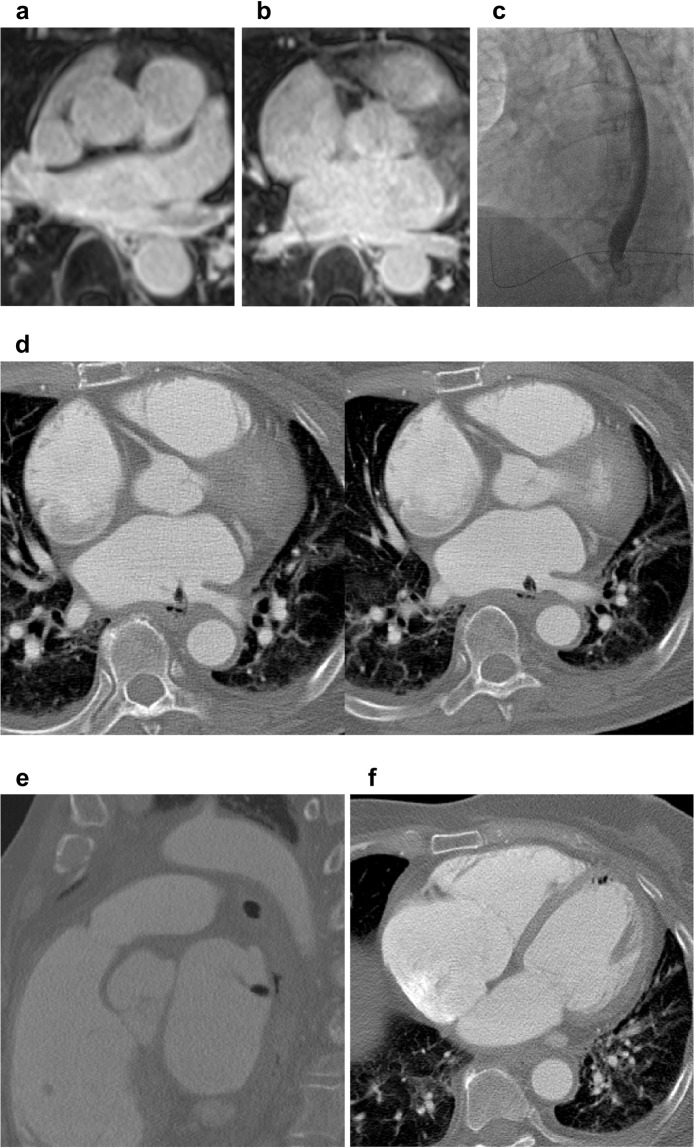
Atrio-esophageal fistula with massive air leakage. (**a–c**) Pre-procedure imaging studies revealed that esophagus was immediately adjacent to left inferior pulmonary vein. (**d,e**) CT scan revealed a definite atrio-esophageal fistula near left inferior pulmonary vein and significant amount of air leakage. (**f**) Free air was also observed in the left ventricle. CT: computed tomography.

**Figure 3:**
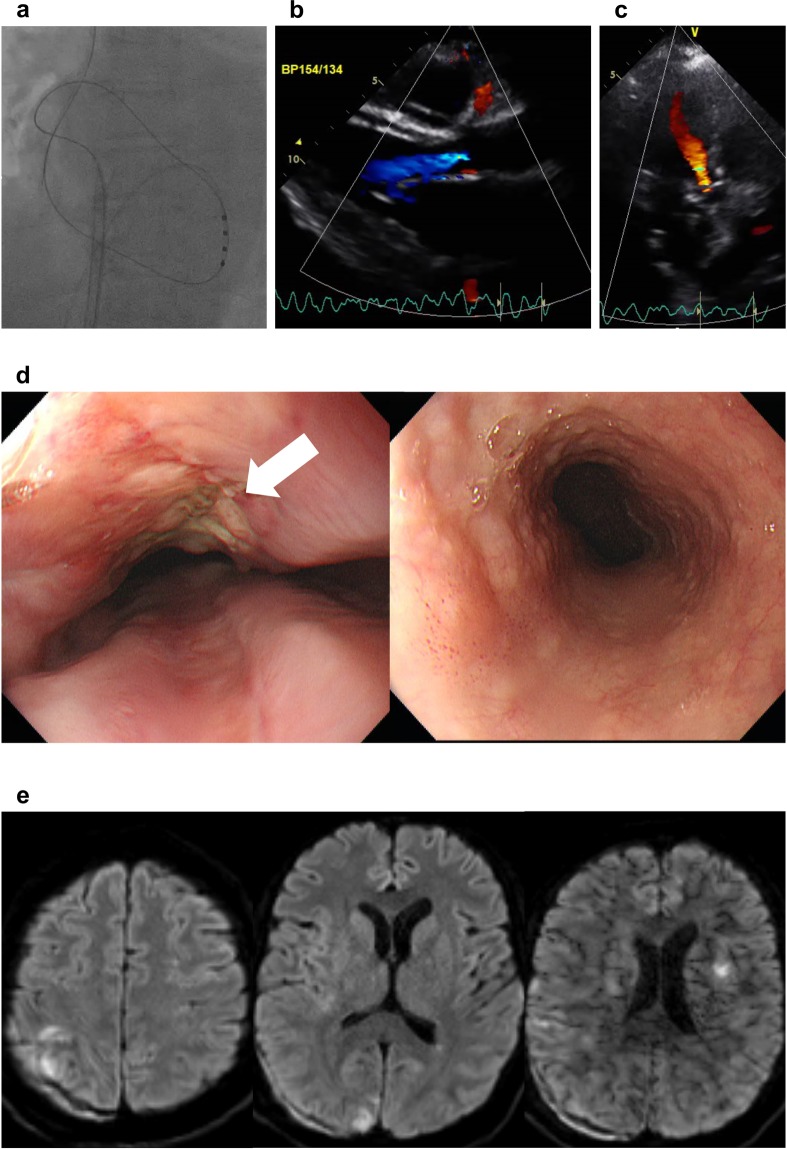
Prevention of embolic stroke and successful surgical repair. (**a**) Ventricular fibrillation was induced using quadripolar catheter immediately after veno-arterial extracorporeal membrane oxygenation apply. (**b,c**) Echocardiography revealed continuous aortic regurgitant jet suggesting complete reversal of blood flow direction in the thoracic aorta. (**d**) On-pump, open heart surgical repair of left atrium and esophagus was performed and follow-up endoscopy revealed no fistula. White arrow indicates operation scar. (**e**) Despite several small-sized embolic lesions, the patient was free of neurologic consequences.

Patient 5 had a large LA defect and massive air leakage was observed in the CT scan (Fig. 2). The operation room was not available at that time. Veno-arterial extracorporeal membrane oxygenation (VA-ECMO) was applied to the patient and ventricular fibrillation (VF) was induced artificially to minimize air and septic embolism in brain (Fig. 3). Mean arterial blood pressure was well maintained and transthoracic echocardiography (TTE) revealed continuous aortic regurgitant jet indicating successful reversal of blood flow in the aorta (Supplementary Video 2–4). After successful repair of the LA, VF was successfully terminated and follow-up TTE performed just before discharge showed slightly improved left ventricular ejection fraction (30% → 35%). The patient had a few small size of cerebral infarction lesions on brain MRI but she was free of neurologic consequences (Fig. 3). The overall results of the five patients according to surgical or endoscopic repair methods are summarized in Table 2. Endoscopy performed 30 days after surgery of the patient 4 and 5 revealed healed AEF (Supplementary Fig. S1 and Fig. 3d).

**Table 2:** Clinical course of each patient.

	Patient 1	Patient 2	Patient 3	Patient 4	Patient 5
Repair type	Surgical	Endoscopic	Surgical	Surgical	Surgical
Esophageal repair	Primary repair with intercostal muscle flap	N/A	Primary repair with intercostal muscle flap	Primary repair with intercostal muscle flap	Primary repair with intercostal muscle flap
Left atrial repair	External repair	N/A	LA repair was not done	• External repair • No bovine pericardium support	• Internal repair • Bovine pericardium support • Open-heart surgery
Redo surgery	No	N/A	No	• Due to infective endocarditis • Internal repair • Bovine pericardium support • Open-heart surgery	No
Diet permission	• Post-operation day 16 • Without endoscopic confirmation	Not permitted	Expired before permission	• Post-operation day 30 • After endoscopic confirmation	• Post-operation day 30 • After endoscopic confirmation
Survival	Expired	Expired	Expired	Survived	Survived
Cause of death	Uncontrolled infection	Uncontrolled infection and cachexia	Sepsis and massive cerebral air embolism	N/A	N/A
VA-ECMO	Not done	Not done	Not done	Not done	Performed before surgery
Neurologic sequela	N/A	N/A	N/A	Right arm weakness and partial aphasia	None

N/A: not applicable; VA-ECMO: veno-arterial extracorporeal membrane oxygenation.

## Discussion

Our case series suggest that (i) timely surgical correction can save patients with AEF; (ii) median sternotomy, on-pump open heart surgery, and LA repair from the inside of the heart seems to be a reasonable surgical approach; (iii) pre-operative VA-ECMO or ventricular assist device application with artificially induced VF might prevent cerebral air and septic embolism through complete reversal of the direction of flow in thoracic aorta. Vigorous studies are conducted to identify certain patient group susceptible to RFCA-related AEF and to find a way to prevent AEF. However, less is known regarding how to overcome such dismal complication. We report five cases which gives us insights on how to overcome AEF caused by catheter ablation of AF and to save patients without neurologic consequences.

### Surgical approach

We consider that aggressive and timely surgery is the cornerstone of the AEF management^16–19^. We had four surgical cases where surgeons confirmed that there was a significant area of thermal injury in both LA and esophagus adjacent to the fistula location. Due to this tissue necrosis, natural healing of the fistula is unlikely^14^. Clinical course of patient 2 in our study also suggest that endoscopic repair of the fistula is neither feasible nor successful. Furthermore, gas injection is performed during endoscopy to inflate esophagus and stomach and this might aggravate systemic air embolism. Patient 3 had no obvious free air in LA but sudden mental retardation occurred during endoscopy suggesting micro-perforation in LA. Endoscopy during acute phase of AEF can be done with CO_2_ gas, but should be avoided whenever possible. Another lesson learned from patient 3 is that absence of LA free air in CT examination does not completely rule out the presence of AEF. If clinical features are suggestive of AEF, vigorous search for LA perforation site can be helpful.

In this study, three patients who had LA repair from the external side all died or underwent re-operation. In patient 5, large amount of foreign materials were found inside LA which was removed, irrigated, and then sutured. The patient recovered without additional surgery. Patient 4 underwent simple repair of LA from the external side but extensive vegetation developed in the sutured site. During re-operation, vegetation was removed from inside of LA and perforation was not healed. Primary repair of LA was performed from internal side of LA and LA perforation was finally healed. Primary repair of LA was performed from the external side in patient 1 and the perforation of LA probably did not completely heal since he suffered uncontrolled sepsis just after ingestion of liquid diet 16 days after the operation. In patient 3, there might have been unrecognized LA micro-perforation during off-pump surgery. Open heart surgery could have been a better option to search for the perforation site by looking at LA from the internal side.

### Prevention of embolism

Cerebral air or septic embolism are major adverse events in patients suffering AEF^16,20,21^. Substantial proportions of embolic events occur in acute phase of AEF and neurologic symptoms related with cerebral embolism are often the presenting symptoms. Currently, there is no established treatment to prevent these embolic complications. Since embolic materials originate from the esophagus and LA perforation site, elevating LA filling pressure or reversing the direction of the blood flow in thoracic aorta might prevent embolic complications. By inducing VF with VA-ECMO application, we were able to completely reverse the direction of the blood flow in the thoracic aorta which was demonstrated by continuous aortic regurgitant jet in TTE evaluation. Although we were not able to directly measure LA pressure, it is well documented that VA-ECMO in a failing heart is associated with significantly increased LA pressure which is paradoxically a good phenomenon in patients with AEF^22,23^. Patient 5 who underwent this maneuver survived without neurologic consequences despite several small-size embolic lesions on brain MRI. The clinical implication of artificial VF induction with simultaneous VA-ECMO application should be evaluated in future trials.

## Limitations

We acknowledge that our study has several limitations. First, this was a retrospective analysis and the number of patients was limited. No statistical comparison was possible due to limited sample size. Second, our cohort is exclusively composed of East Asian people and it is unclear whether our results can expand to other ethnic groups. Third, although our study suggests potentially effective method to treat AEF, how to prevent AEF remains unclear. However, our previous study identified certain risk factors for AEF such as old age, low body weight, and high CHA_2_DS_2_-VASc score^7^. Severity of endoscopically detected esophageal thermal injury was also reported to be a predictor of AEF^24^. Recent study suggests that high-resolution infrared thermal imaging can effectively predict endoscopically detected thermal esophageal lesions^25^. Halbfass *et al*. evaluated the impact of ablation index-guided approach in terms of esophageal thermal injury and found that the incidence of esophageal thermal injury was slightly lower than expected from previous studies^26^.

## Conclusions

Sequential repair of LA and esophagus can facilitate the healing of AEF. On-pump, open heart surgical repair of LA with bovine pericardium support might be an appropriate surgical approach. Primary esophageal repair with intercostal muscle flap interposition seems to be sufficient. If clinical situation is compatible with AEF, searching for LA perforation from the inside of the heart might be necessary even in patients without obvious free air in LA in CT scan. Artificial induction of VF and simultaneous application of VA-ECMO might prevent serious cerebral embolic events.

## Supplementary information

Supplementary information.

Supplementary Figure S1. 

Supplementary Video 1.

Supplementary Video 2.

Supplementary Video 3.

Supplementary Video 4.

## Data Availability

The data analyzed during the current study are available from the corresponding author on reasonable request.
