# Supplementary figures and images for: Management of Atrio-esophageal Fistula Induced by Radiofrequency Catheter Ablation in Atrial Fibrillation Patients: a Case Series
Source: Sci Rep. 2020 May 18;10:8202. doi: 10.1038/s41598-020-65185-9 (PMC7235255; doi:10.1038/s41598-020-65185-9)

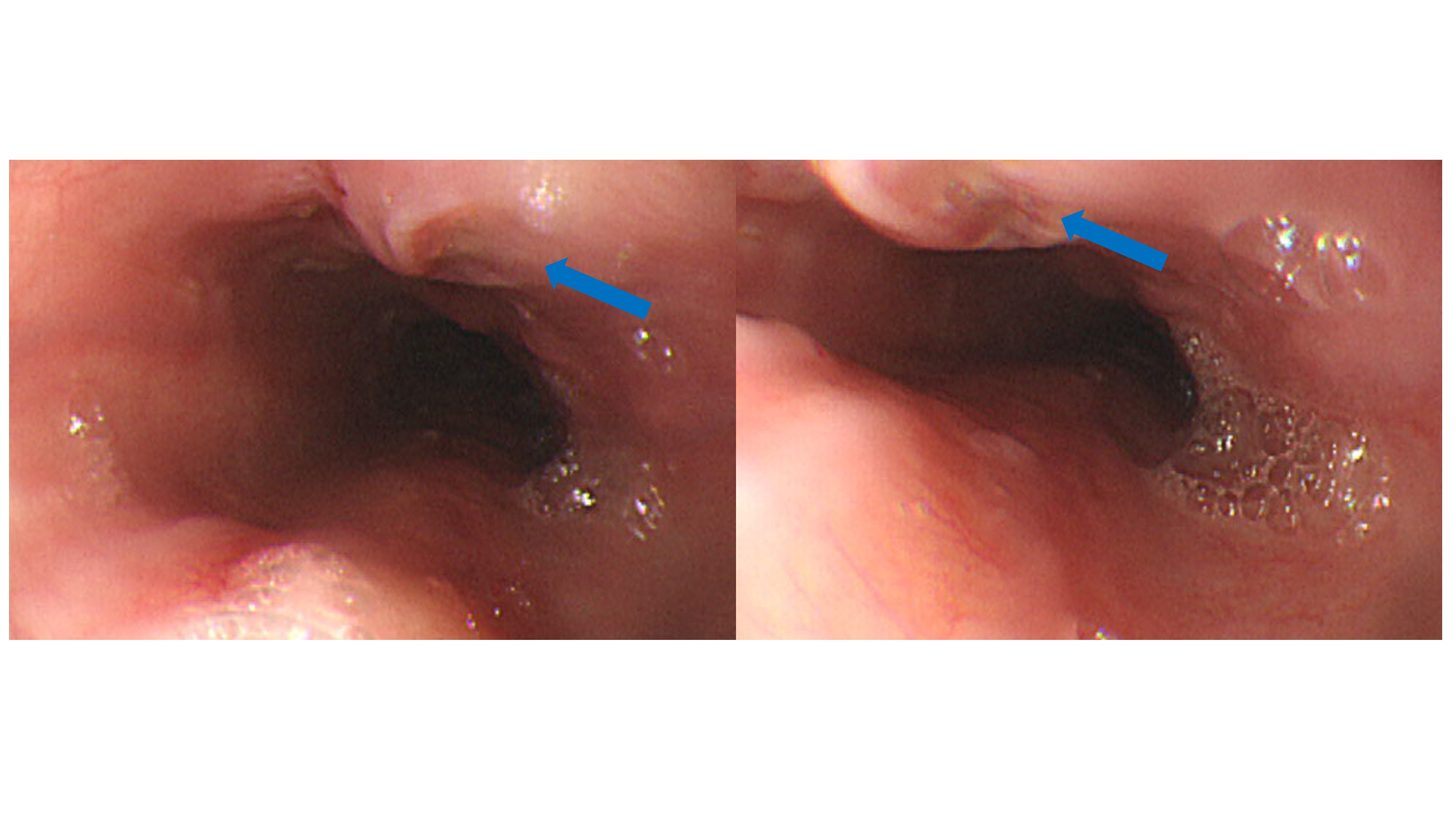

Supplement: Supplementary file 1 — Supplementary information. [file 41598_2020_65185_MOESM1_ESM.jpg]
