# Supplementary material for: Management of Atrio-esophageal Fistula Induced by Radiofrequency Catheter Ablation in Atrial Fibrillation Patients: a Case Series
Source: Sci Rep. 2020 May 18;10:8202. doi: 10.1038/s41598-020-65185-9 (PMC7235255; doi:10.1038/s41598-020-65185-9)
Supplement: Supplementary file 2 — Supplementary Figure S1. [file 41598_2020_65185_MOESM2_ESM.docx]

**Management of Atrio-esophageal Fistula Induced by Radiofrequency Catheter Ablation in Atrial Fibrillation Patients: a Case Series**

Yun Gi Kim, MD,^1^ Jaemin Shim, MD,^1*^ Kwang-No Lee, MD,^1^ Ju Yong Lim, MD,^2^ Jae Ho Chung, MD,^2^ Jae Seung Jung,^2^ MD, Jong-Il Choi, MD,^1^ Sung Ho Lee, MD,^2^ Ho Sung Son, MD,^2^ and Young-Hoon Kim, MD^1*^

^1^: Arrhythmia Center, Korea University Medical Center Anam Hospital, Seoul, Republic of Korea

^2^: Department of Thoracic and Cardiovascular Surgery, Korea University Medical Center Anam Hospital, Seoul, Republic of Korea

* **Correspondence**

Jaemin Shim, MD, PhD

Arrhythmia Center, Korea University Medical Center Anam Hospital

73, Inchon-ro, Seongbuk-gu, Seoul 02841, Republic of Korea

Tel: 82-2-920-5445

Fax: 82-2-927-1478

E-mail: [jaemins@korea.ac.kr](mailto:jaemins@korea.ac.kr)

Young-Hoon Kim, MD, PhD

Arrhythmia Center, Korea University Medical Center Anam Hospital

73, Inchon-ro, Seongbuk-gu, Seoul 02841, Republic of Korea

Tel: 82-2-920-5211

Fax: 82-2-923-6030

E-mail: yhkmd@korea.ac.kr

**Running title:** How to survive atrio-esophageal fistula

**Words count:** 3,501

**Key words:** Atrial fibrillation; Atrio-esophageal fistula; Radiofrequency catheter ablation.

**Supplementary Figure Legends**

**Figure S1.** Endoscopic finding of healed AEF.

Endoscopy was performed in patient 4 30 days after re-do surgery. Previous AEF site was healed.

**Supplementary Video Legends**

**Supplementary Video 1.** LA vegetation.

LA vegetation was observed after LA repair from external side without bovine pericardium patch support.

LA: left atrium.

**Supplementary Video 2.** VA-ECMO and VF induction.

Parasternal long axis view shows no systolic motion in a patient with VA-ECMO application and VF induction.

VA-ECMO: veno-arterial extracorporeal membrane oxygenation; VF: ventricular fibrillation.

**Supplementary Video 3.** Flow reversal: parasternal long-axis view.

Parasternal long-axis view with color Doppler shows continuous aortic regurgitant jet suggesting flow reversal in the aorta in a patient with VA-ECMO application and VF induction.

VA-ECMO: veno-arterial extracorporeal membrane oxygenation; VF: ventricular fibrillation.

**Supplementary Video 4.** Flow reversal: apical view.

Apical view with color Doppler shows continuous aortic regurgitant jet suggesting flow reversal in the aorta in a patient with VA-ECMO application and VF induction.

VA-ECMO: veno-arterial extracorporeal membrane oxygenation; VF: ventricular fibrillation.
